# Comparison of the efficacies of TINAVI robot-assisted surgery and conventional open surgery for Levine–Edward type IIA (postreduction) hangman fractures

**DOI:** 10.1038/s41598-023-43136-4

**Published:** 2023-09-23

**Authors:** Shuai Li, Jinpeng Du, Yunfei Huang, Dingjun Hao, Zhigang Zhao, Zhen Chang, Jingwen Zhu, Xiaodong Wang, Yongchao Duan, BaoRong He

**Affiliations:** 1 Department of Spine Surgery, Honghui Hospital, Xi’an Jiaotong University, Youyidong Road, Xi’an, 710000 Shaanxi China; 2 Medical College, Yan’an University, Yan’an, Shaanxi China

**Keywords:** Spinal cord diseases, Bone

## Abstract

The objective was to compare the clinical efficacy of percutaneous pedicle screw internal fixation with the aid of the TINAVI orthopaedic surgery robot with that of traditional open surgery for Levine–Edward type IIA (postreduction) hangman fractures and to evaluate the safety and efficacy of the TINAVI robot-assisted orthopaedic surgery procedure. The clinical data of 60 patients with Levine–Edward type IIA (postreduction) hangman fractures treated surgically from June 2015 to February 2022 were analysed retrospectively. Among these patients, 25 were treated with percutaneous pedicle screw fixation under TINAVI (the robot group), and 35 were treated with pedicle screw implantation assisted by a conventional C-arm X-ray machine (the traditional operation group). The pedicle screw placement grade was evaluated according to the Rampersaud scale. The correct rate of pedicle screw placement was calculated. The invasion of adjacent facet joints, VAS score (Visual Analogue Scale), NDI score (Neck Disability Index), SF-36 score (36-Item Short-Form Health Survey questionnaire), EQ-5D score (EuroQol-5 dimensions questionnaire) and operation-related data were recorded, and patients were followed up. All patients were followed up for an average of 15.0 ± 3.4 months. The accuracy of screw placement in the robot group was higher than that in the traditional operation group, while the rates of intraoperative blood loss and invasion of the facet joint were lower and the incision length and length of hospital stay were shorter. On the 3rd day after the operation, the VAS score in the robot group was significantly higher than that in the traditional operation group, but there was no significant difference in the NDI score. On the 3rd day after the operation, the SF-36 and EQ-5 questionnaire scores of the robot group were better than those of the traditional operation group. No complications occurred in any of the patients. Postoperative cervical X-ray showed that the cervical vertebra was stable, and there was no fracture, angle or displacement. Postoperative CT showed that all fractures healed, and the average healing time was 3.4 months. The treatment of Levine–Edward IIA (postrepositioning) hangman fractures with percutaneous pedicle fixation assisted by the TINAVI orthopaedic surgery robot can significantly improve screw placement accuracy with a low rate of invasion of the adjacent facet joint, a short operation time, a low bleeding rate, and high patient satisfaction. Although there are still many disadvantages, it still has good prospects for application.

## Introduction

Hangman fractures, also known as traumatic pivotal slippage, are bilateral C2 pivotal arch or interarticular fractures with or without anterior C2 dislocation, accounting for 4–7% of all cervical fractures and 20–22% of all pivotal fractures; these fractures are the second most common injury to the pivotal spine^1^. Fractures may extend into the posterior cortex of the vertebral body or into the vertebral artery foramen, but most patients with vertebral artery injury are clinically asymptomatic. This type of fracture is usually seen in hyperextension and axial loading and sometimes in flexion injuries^2^. Such fractures are usually the result of high-energy trauma and are commonly seen after motor vehicle accidents, falls from height, and objects falling from a great height. The treatment strategy for hangman fractures is based on the Levine and Edwards classification of fracture stability^3^. Patients with stable type I injuries can be treated nonoperatively with halo undershirts, Minerva jackets, and stiff neck collars; pseudarthrosis formation, anterior C2 dislocation, C2–3 entrapment, and recurrent axial pain occur in approximately 60% of patients who are treated conservatively for unstable type II, IIA, and III fractures^4^. Most scholars believe that it is difficult to obtain closed reduction for unstable hangman fractures due to posterior wall defects of the vertebral body, fracture fragments and soft tissue impaction. Patients with unstable hangman fractures can usually benefit from anterior cervical nucleus pulposus fusion (ACDF), posterior fixed fusion, pedicle screws alone or a combined anterior–posterior approach^5^. In recent years, with the vigorous development of medical navigation technology, the accuracy and safety of orthopaedic robot-assisted surgeries have been continuously improved, especially in C2 posterior pedicle screw internal fixation, which has shown the advantages of high accuracy and minimal invasiveness. TINAVI is a 3rd generation orthopaedic surgery robot that was developed independently in China and is the only robot that has been awarded China Food and Drug Administration approval for spinal surgery, which has greater advantages in minimally invasive internal fixation for fresh nondisplaced navicular fractures and complex pelvic acetabular fractures^6^. The purpose of this study was to report the imaging and clinical outcomes of TINAVI orthopaedic robot-assisted transpedicular pedicle screw fixation for hangman fractures.

## Clinical data and methods

### General data

All methods were performed in accordance with the Strobe Statement guidelines, and was conducted in accordance with the Declaration of Helsinki.

This retrospective study was approved by the Ethics Committee of Honghui Hospital, Xi’an Jiaotong University (approval number: 20220014).

Informed consent has been obtained from all subjects and/or their legal guardians.

**Figure 1:**
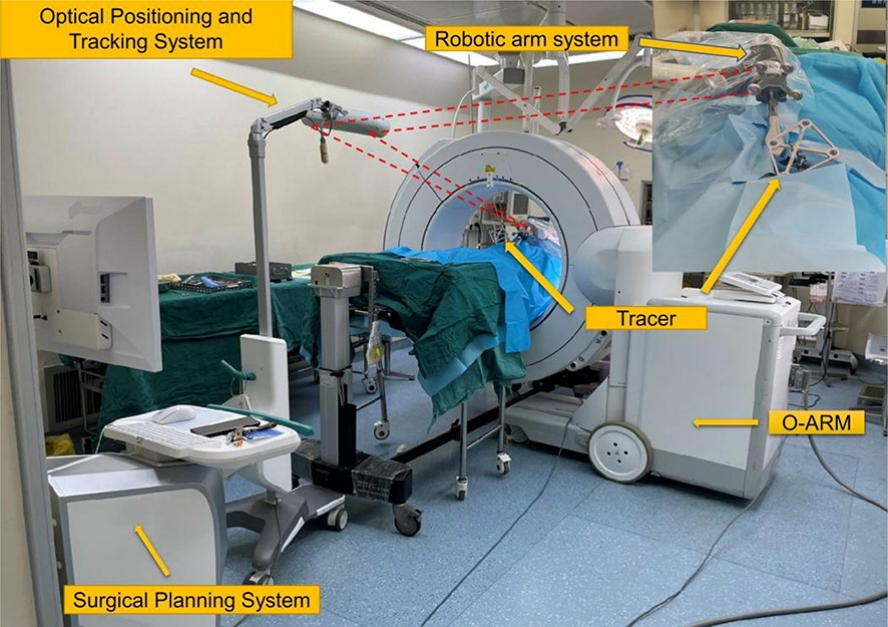
TINAVI orthopaedic robot structure and placement.

From June 2015 to March 2019, we mainly used traditional C-arm X-ray machines to treat Levine–Edward type IIA (postreduction) hangman fractures. In March 2019, with the introduction of the TINAVI orthopaedic robot, we began to use a robot system to treat patients with Levine–Edward type IIA (postreduction) hangman fractures. The clinical data of the surgical methods were analysed retrospectively. (Fig. 1).

#### Inclusion criteria

① Patients with mild to moderate displacement (horizontal displacement of the fracture block < 5 mm, C2 and C3 at an angle < 15°) of typical or atypical Levine–Edward type IIA hangman fractures (combined with longitudinal splitting of the posterior edge of the C2 vertebral body); ② patients without nerve or vertebral artery injuries (nerve injury was determined by systemic sensory, motor-related examinations and electromyography. Vertebral artery injury was diagnosed by MR angiography); ③ patients aged 15–80 years; ④ patients with a clear history of trauma.

#### Exclusion criteria

① Patients with serious medical diseases or multiple injuries who could not tolerate surgery; ② patients with nerve and vascular injuries that required open surgery for exploration and decompression; ③ patients lost to follow-up within 3 months after surgery; ④ patients with congenital malformations, anatomical variations and vertebral foramen malformations in the pedicle; and ⑤ patients with a bone mineral density < − 2.5 and a history of osteoporotic fractures.

### Surgical method

After admission, according to the X-ray film of the cervical vertebra before traction, small weight skull traction forceps were used to gently grip the cervical vertebra along the force line to avoid excessive extension and flexion traction and gradually increase the weight so that the displaced fracture block was realigned with C2–3 in the sagittal plane. Lateral X-ray films of cervical vertebrae were taken 4–6 h after traction to evaluate the degree of cervical rearrangement.

#### Robot group

**Figure 2:**
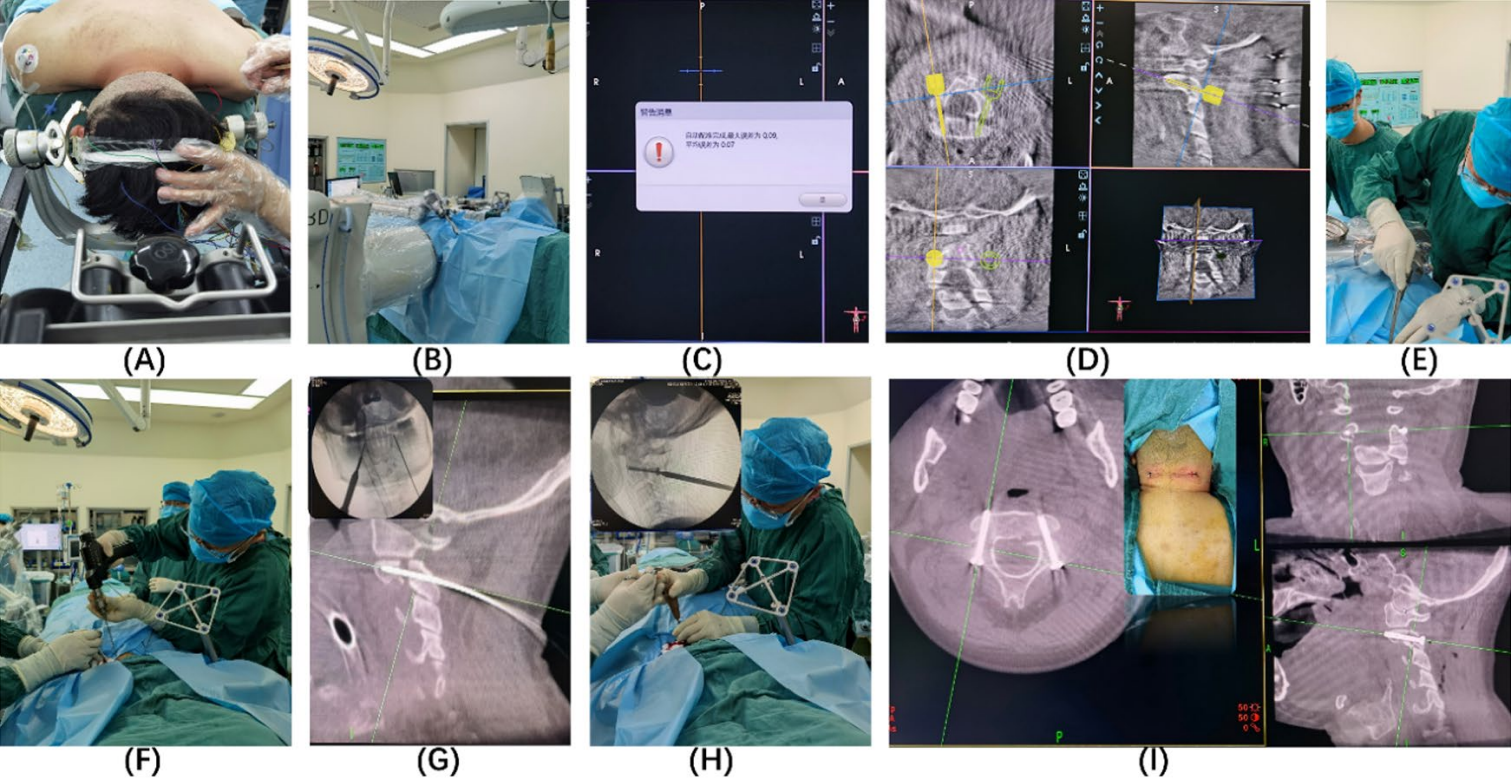
(**A**) Head frame fixation (**B**) Placement of the TINAVI orthopaedic robot (**C**) Acquisition and configuration (**D**) Planning of needle entry point and needle direction (**E**) Incision at the skin entry point and blunt separation to isolate the subcutaneous tissue (**F**) Placement of the guide needle (**G**) Verification of the guide needle position (**H**) Screw placement (**I**) Verification of the screw position.

After general anaesthesia, the patient was placed in the prone position, head suspension was performed, Mayfield head frame was used to fix the head, skull traction was performed, the traction weight was adjusted, and X-ray film was taken to determine the basic condition of the patient and the changes in the sensory muscle strength of the limbs at any time. It was necessary to pay attention to the changes in ECG monitoring at all times. If there was a decrease in blood pressure, heart rate, finger pulse oxygen saturation or other indices, traction weight was reduced or traction was stopped in a timely manner. Fluoroscopy was performed using a C-arm X-ray machine with the C2 vertebra centrally located, and after confirming satisfactory fracture repositioning, the various components of the TINAVI orthopaedic surgical robot, including the optical tracking system, the surgical planning and navigation system, and the robotic arm system, were connected, and the TINAVI robotic patient tracer was mounted on a bedside stand. The surgical area was routinely disinfected, and sterile sheets were laid. The patient's cervical spine was scanned circumferentially using an O-arm CT machine (Medtronic) centred on the pivot vertebrae, and the image data were transmitted to the TINAVI robotic table to plan the bilateral pedicle screw trajectory of the pivot vertebrae, avoiding the spinal cord and vertebral artery as much as possible. The arm of the TINAVI robot was set at the designated position, and 2 positioning guidewires were placed with the help of a high-speed electric drill under a condition of deviation less than 1 mm. Fluoroscopy and CT scans were performed to determine whether the positioning guidewires were in a good position, and a hollow drill was used to drill the screw path along the guidewires. Two hollow tension screws were placed, and 2 guidewires were removed. The wound was irrigated and observed to determine whether there was active bleeding, and the instrument dressings were counted. The wound was sutured and covered with a sterile dressing. Murphy's head frame was removed, and the neck was braced with a neck brace, which indicated the end of surgery (Fig. 2).

#### Traditional surgery group

Patients were placed in a prone position after general anaesthetic intubation with head pad protection. A sterile towel sheet was placed and disinfection and affixing of the incision film were performed according to the posterior cervical surgery routine with Anl iodine. A posterior median cervical incision approximately 10 cm long was made, starting from the occipital eminence and ending at the cervical 2 spinal eminence. The paravertebral muscles were bluntly dissected to reveal the posterior arch of the atlantoaxial spine and the spinous process of the pivot. The pedicle screws were fixed at both sides of the atlantoaxial spine. C-arm fluoroscopy showed that the screws were well positioned and that the fracture was well fixed. One drainage tube was placed in the wound, the number of dressings and instruments was counted correctly, and the neck wound was closed layer by layer. A sterile dressing was applied with pressure. A neck brace was placed, and the operation was completed.

### Postoperative treatment

Antibiotics and haemostatic drugs were routinely used within 24 h after the operation. From the second day after the operation, patients were encouraged to wear cervical spine stents to get out of bed. The cervical X-ray film was reexamined 3 days after the operation, the cervical stent was removed 3 months after the operation, and cervical functional exercise was actively carried out. The positive and lateral X-ray films of the cervical vertebra were examined regularly at 1, 3, 6, 9 and 12 months after the operation. A thin-slice CT scan was performed 3 days after the operation to evaluate the fusion of the implants, to observe whether the cervical height and curvature had been lost again, to observe whether the internal fixed screws had loosened and whether operative complications had occurred.

### Evaluation indicators

#### Main indicator

##### Screw insertion accuracy

All patients underwent a postoperative CT examination, and postoperative CT image data were measured using the Picture Archiving and Communication System (PACS). The measurement of pedicle screw position was independently assessed by two spine surgeons who were not involved in the surgical procedure. Disputes were resolved by deliberation. The accuracy of screw placement was assessed according to the Rampersaud scale^7^: Level 0: screws were completely within the pedicle; Level 1: screws penetrated < 2 mm into the pedicle cortex; Level 2: screws penetrated < 4 mm into the pedicle cortex; and Level 3: screws penetrated ≥ 4 mm into the pedicle cortex. Level 0 was considered the “ideal screw position”, Levels 1 + 2 were considered “clinically acceptable” screw placement, and Levels 3 and 4 were considered “unacceptable” screw placement.

##### Invasion of adjacent facet joints

Invasion of the adjacent facet joint was evaluated according to the classification described by Kim et al.^8^: Level 0 = no contact, Level 1 = screw head contact or suspected contact with the small joint, and Level 2 = the screw clearly invades the small joint.

#### Secondary indicators

The operation time, single nail placement time, intraoperative blood loss, intraoperative fluoroscopic dose, incision length and length of hospital stay were compared between the two groups.

The VAS score and NDI score before the operation, 3 days after the operation, 3 months after the operation and at the last follow-up were compared between the two groups.

Two sets of functional outcome indicators, such as the 36-item Short-form health survey questionnaire and the EuroQol five dimensions questionnaire, were compared.

The cost of using the equipment was compared between the two groups.

Complications such as screw loosening or pullout, infection, iatrogenic neurovascular injury and posterior cervical haematoma were compared between the two groups.

SF-36 (36-item Short-form health survey questionnaire): This is a very popular questionnaire used to evaluate health-related quality of life. It comprises 8 sections: physical function, occupational-related physical factors, general health status, a physical pain score, a social ability assessment, occupational-related mental factors, a mental state assessment and a mental health score.

EQ-5D questionnaire (EuroQol five dimensions questionnaire): This is a general health status measurement tool that was developed by the EuroQol group. It evaluates the health status of the population in the form of a questionnaire and describes the quality of life. It mainly includes five dimensions: activity ability, self-care, daily activities, pain/discomfort, and anxiety/depression. Each dimension is divided into three levels: no difficulties, some difficulties and extreme difficulties.

### Statistical analysis

Data were analysed using SPSS 25.0 statistical software. Data are expressed as the mean ± standard deviation $(\overline{x} \pm s)$. Preoperative and follow-up outcomes were compared using paired t tests if all data passed the Shapiro‒Wilk normality test. Differences between the two groups were compared using the independent samples t test. A one-tailed test was adopted. Differences in general information and clinical outcomes were compared using the chi-square test. *P* < 0.05 was considered statistically significant.

## Results

As shown in Table 1, a total of 60 patients met the criteria and were included in the robot group (n = 25) and traditional surgery group (n = 35), with a male-to-female ratio of 41 to 19 and an average age of 43.2 ± 11.5 years. All patients were followed up for an average of 12.6 months. Fifty screws were implanted in the TINAVI robot group, and 70 screws were implanted in the traditional surgery group. The screw diameter was 3.5 mm. One segment was fixed in all patients. All patients had varying degrees of neck pain, but their neurological function was normal. Regarding injury factors, most patients had head and neck hyperextension due to violence. All patients completed cervical DR and CT + 3D reconstruction before the operation, and MRI examination was performed to determine the type of fracture, soft tissue injury and location of the vertebral artery. The main diagnosis of this group of patients was Levine–Edward type IIA hangman fracture. There was no significant difference in the baseline data between the two groups (*P* > 0.05).

**Table 1:** Baseline characteristics.

Indicators	Robot group	Traditional surgery group	t/Z/χ^2^	P
	N = 25	N = 35		
Sex				
Male	17	24	0.000	0.963
Female	8	11		
Age (years)	44.3 ± 12.6	42.4 ± 10.6	0.633	0.529
Follow-up time (months)	15.7 ± 4.2	14.6 ± 2.6	1.160	0.253
Type of injury				
Traffic accident	10	21	3.70	0.157
Fall from height injuries	14	11		
Injuries from falling objects	1	3		
Time from injury to hospitalization (days)	3.9 ± 1.3	3.7 ± 1.1	0.644	0.522
C2-3 injury				
Anterior longitudinal ligament	20	6	4.12	0.127
Posterior longitudinal ligament	25	18		
Intervertebral disc	6	7		
C2-3 Clamping angle and displacement				
Angle (°)	10.5 ± 3.5	11.7 ± 3.5	1.31	0.196
Displacement (mm)	3.5 ± 1.3	3.4 ± 1.6	0.26	0.798
Combined with other vertebral injuries	0	3		

Analysis by independent samples t test. Comparison between the two groups, *p* < 0.05.

### Accuracy of pedicle screw placement

The accuracy of “perfect” and “clinically acceptable” pedicle screw placement ranged from 88 to 98% in the TINAVI group and from 53 to 74% in the traditional surgery group (Table 2), with significant differences between the two groups (*P* < 0.05).

**Table 2:** Implant screw rating and comparison.

Grading of pedicle screw positioning	Robot group	Traditional surgery group	t/Z/χ^2^	*P*
Level 0	44	37	4.03	< 0.001
Level 1	5	15		
Level 0 + 1	49	52	2.36	0.018
Level 2	1	15		
Level 3	0	3		

Analysed by chi-square test, *P* < 0.05.

### Invasion of adjacent facet joints

Regarding invasion of the adjacent facet joint, there was a significant difference between the TINAVI group (grade 0–2: 96.0%, 4%, 0%) and the traditional surgery group (grade 0–2: 60%, 40%, 0%) (*P* < 0.05) (Table 3).

**Table 3:** Comparative results of the invasion of adjacent facet joints.

Indicators	Robot group	Traditional surgery group	t/Z/χ^2^	*P*
Invasion of the adjacent facet joint				
Level 0	48	42	3.89	< 0.001
Level 1	2	28		
Level 2	0	0		

Analysed by chi-square test, *P* < 0.05.

### Clinical results

The operation time, single nail implantation time (from the skin incision to the completion of implantation), incision length and hospital stay were shorter and the intraoperative blood loss rate was lower in the TINAVI group than in the traditional surgery group (*P* < 0.05). The intraoperative fluoroscopic dose in the TINAVI group was significantly higher than that in the traditional surgery group (*P* < 0.05) (Table 4).

**Table 4:** Perioperative clinical indicators.

Indicators	Robot group	Traditional surgery group	t/Z/χ^2^	*P*
Surgery time (min)	97.0 ± 10.5	150.7 ± 13.8	16.353	< 0.001
Single screw implantation time (min)	2.5 ± 0.2	4.2 ± 0.1	39.148	< 0.001
Intraoperative bleeding (ml)	40.7 ± 15.8	189.9 ± 128.2	6.813	< 0.001
Intraoperative fluoroscopic dose (mGy)	256.0 ± 42.3	36.73 ± 8.57	25.547	< 0.001
Incision length (cm)	2.0 ± 0.3	10.0 ± 1.2	37.821	< 0.001
Length of hospitalization (days)	3.9 ± 0.6	6.3 ± 2.1	6.405	< 0.001

Definition of the single screw placement time: after adequate exposure, from the beginning of the determination of the needle entry point to the end of screw placement.

Analysis by independent samples t test. Comparison between the two groups, *P* < 0.05.

The VAS score at 3 days after the operation was lower in the TINAVI group than in the traditional surgery group (*P* < 0.05) (Table 5).

**Table 5:** VAS Score.

VAS	Robot group	Traditional surgery group	t/Z/χ^2^	*P*
Preoperative	7.9 ± 0.9	8.3 ± 1.1	1.495	0.140
3 Days after surgery	1.3 ± 0.4^a^	3.4 ± 0.5^a^	17.386	< 0.001
3 Months after surgery	1.1 ± 0.5^a^	1.2 ± 0.4^a^	0.860	0.393
Last follow-up visit	1.0 ± 0.2^a^	1.1 ± 0.3^a^	1.548	0.127

*VAS* visual analogue scale.

Analysed by paired t tests, compared with before the operation, *P* < 0.05.

Analysis by independent samples t test. Comparison between the two groups, *P* < 0.05.

^a^represents a statistically significant difference preoperatively compared to postoperatively.

The NDI score at 3 days after the operation was lower in the TINAVI group than in the traditional surgery group (*P* < 0.05) (Table 6).

**Table 6:** NDI Score.

Indicators	Robot group	Traditional surgery group	t/Z/χ^2^	*P*
NDI Score				
Preoperative	32.6 ± 6.3	35.4 ± 8.3	0.101	0.920
3 Days after surgery	15.2 ± 3.3^a^	14.4 ± 4.8^a^	0.720	0.475
3 Months after surgery	10.1 ± 2.1^a^	11.1 ± 1.9^a^	1.924	0.059
Last follow-up visit	7.2 ± 1.3^a^	7.1 ± 3.2^a^	0.167	0.868

*NDI* neck disability index.

Analysed by paired t test, compared with before the operation, *P* < 0.05.

Analysis by independent samples t test. Comparison between the two groups, *p* < 0.05.

^a^represents a statistically significant difference preoperatively compared to postoperatively.

Three days after the operation, the SF-36 and EQ-5D scores were higher in the TINAVI group than the traditional surgery group (*P* < 0.05) (Table 7).

**Table 7:** SF-36 and EQ-5D.

Indicators	Robot group	Traditional surgery group	t/Z/χ^2^	*P*
PCS scores				
Preoperative	41.2 ± 6.1	42.4 ± 6.3	0.703	0.485
3 Days after surgery	49.3 ± 5.1^a^	45.2 ± 6.5^a^	2.646	0.017
3 Months after surgery	51.2 ± 6.9^a^	52.0 ± 8.0^a^	0.382	0.704
Last follow-up visit	52.9 ± 6.9^a^	53.1 ± 6.2^a^	0.113	0.910
MCS scores				
Preoperative	53.8 ± 4.9^a^	55.0 ± 4.8^a^	0.907	0.368
3 Days after surgery	62.9 ± 4.9^a^	61.8 ± 4.2^a^	3.105	0.003
3 Months after surgery	65.2 ± 5.6^a^	65.1 ± 4.8^a^	0.518	0.607
Last follow-up visit	65.4 ± 4.8^a^	65.8 ± 3.9^a^	3.050	0.730
EQ-5D				
Preoperative	0.48 ± 0.16	0.49 ± 0.19	0.606	0.547
3 Days after surgery	0.59 ± 0.06^a^	0.52 ± 0.01^a^	5.182	0.000
3 Months after surgery	0.61 ± 0.09^a^	0.60 ± 0.05^a^	0.462	2.787
Last follow-up visit	0.67 ± 0.1^a^	0.61 ± 0.2^a^	1.549	0.063

*SF-36* 36-item short-form health survey questionnaire, *EQ-5D* EuroQol-5 dimensions questionnaire.

^a^represents a statistically significant difference preoperatively compared to postoperatively.

The cost of each surgery using the TINAVI robot was approximately 3000 yuan, which was significantly higher than that of the traditional surgery (*P* < 0.05). There were no serious complications, such as screw loosening or infection, in the two groups, and there was no significant difference between the two groups (*P* > 0.05).

#### Typical case

**Figure 3:**
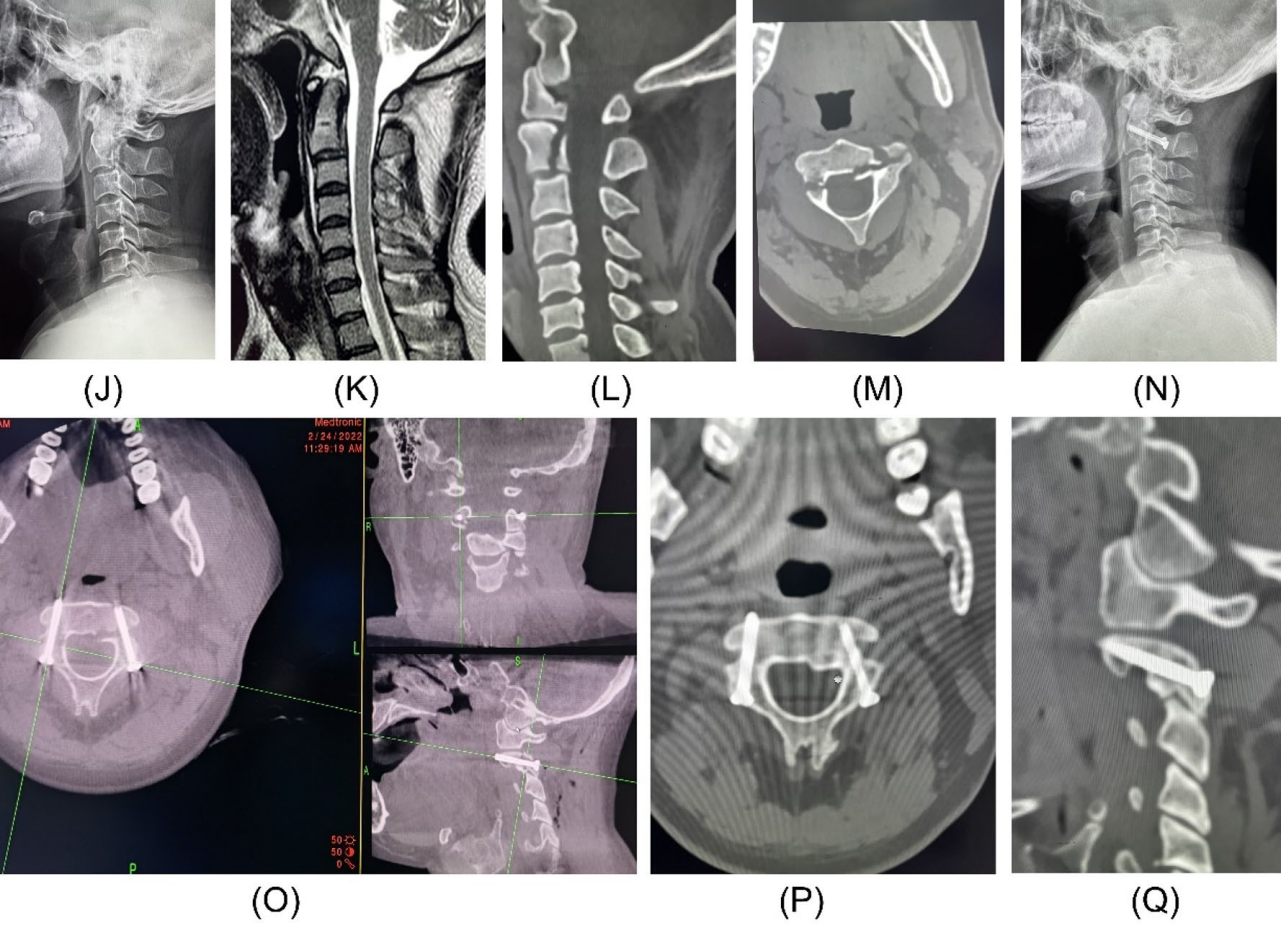
(**J**) preoperative DR, (**K**) preoperative MRI, (**L**–**M**) postoperative CT, (**N**) postoperative DR, (**P**) postoperative CT, (**P**–**Q**) last follow-up CT.

A 40-year-old male patient had fallen, which caused neck pain, and he was diagnosed with hangman fracture. On examination, he had limited left and right rotation of the neck, no radiating pain in the extremities, normal muscle strength of the extremities, low muscle tone, and negative pathological signs. The patient was admitted to the hospital and underwent posterior robot-assisted percutaneous internal fixation of the cervical spine for hangman’s fracture and was given routine treatment, including antibiotics and cervical braces, after surgery. The patient recovered well from the neck and returned to normal life and work (Fig. 3).

## Discussion

Type I hangman fractures are stable injuries and are usually treated conservatively. All other hangman fracture types are unstable injuries and tend to require surgical intervention^9^. Anterior surgery has disadvantages such as complex anatomy and poor stability, while posterior surgery can provide better stability through three-column fixation, with the posterior pedicle screw technique standing out due to its unique low rates of trauma and complications^10^. However, the height of the C2 pedicle is approximately 9–11 mm, and the width is approximately 7–9 mm^11^, with the cervical medulla located medially and the vertebral artery foramen located laterally and inferiorly, and there may be a pedicle variation or high vertebral artery span. Therefore, if one 3.5 mm diameter screw is used for fixation, the screw will have a nearly unique path in the pedicle, and it is extremely difficult to adjust the position if the initial placement is not ideal^12^. Trans-injured spine screw placement may also aggravate fracture displacement and angulation^13^. With the recent boom in digital orthopaedic technology, newer navigation robotic-assisted systems are focusing on this challenge for precise guidance of screw placement.

The TINAVI robot is a new orthopaedic surgical device with human‒machine interaction as the main theme. It consists of a mobile 6-degree-of-freedom robotic arm system, an optical positioning and tracking system, a surgical planning and navigation system, a tracer, and an O-arm^14^. The surgeon plans the screw implantation trajectory based on the surgical operation and planning system in the 3D mode. Subsequently, the robotic arm automatically calculates the spatial position of the planned trajectory and, together with the optical tracking system, moves precisely to the planned position and guides the surgeon in precisely placing the screw through the trocar with the help of a guide pin^15^. The optical tracking system consists of an infrared stereoscopic camera and two tracers (attached to the spinous process and used by the robotic arm for positioning), which cooperate with the robotic arm to achieve real-time motion compensation and ensure that the robotic arm accurately performs the intended trajectory.

The results of this study showed that the shorter screw placement time (2.5 ± 0.2 min) in the TINAVI group compared to the traditional surgery group (4.2 ± 0.1 min) was due to the advance planning of the screw tract. However, patients in the TINAVI group had significantly increased exposure to fluoroscopic doses due to the need for intraoperative 3D image reconstruction by the 3D-O arm. However, the operator received a near-zero radiation dose due to the distance from the operating room before acquisition. Qingqing Li^16^ reported 91.4% accuracy of level 0 screw implants in the TINAVI group, which was similar to our study and significantly different from the accuracy in the traditional surgery group. The TINAVI robot was able to accurately plan the screw path and run smoothly by the operator’s rough placement and precise positioning of the robot’s active position, with the O-arm acquiring information. Intraoperative real-time detection of the inlet and outlet points and timely correction were performed if the deviation was > 0.1 mm. There was a statistically significant difference in the mean operative time in the TINAVI group (97.0 ± 10.5 min) compared with the traditional surgery group (150.7 ± 13.8 min), and multiple pedicle screw placements could be completed at once with robotic assistance and also due to the certain proficiency in the operation of the robot at our institution before the study. Malik et al.^15^ conducted a study on the complications of robot-assisted spine surgery and showed that the incidence of complications was not increased with robotic assistance, which is consistent with our results, and no serious complications occurred in either group. Ruyu Zhao et al.^17^ found that the intraoperative bleeding rate, VAS scores, and NDI scores were lower and the incision length and length of hospital stay were shorter in the TINAVI robotic group than in the traditional surgery group. Lu-Ping Zhou et al.^18^ concluded that precision screw implantation can be robotically assisted in effectively avoiding the medically induced injuries to the adjacent facet joint during screw implantation.

Robot-assisted spine surgery, except for precision screw fixation, expands the application in numerous directions. The separation of the anterior and posterior structures of the cardinal spine after fracture contributes to an increase in the sagittal diameter of the spinal canal to achieve indirect decompression, and some patients have insignificant clinical symptoms and missing C2 pedicles due to chronic fracture and resorption^19^. In these patients, the operator usually chooses to place screws directly into the vertebral body, and preoperative planning of the screw tract can effectively guide the reconstruction of the pedicle^20^. There are numerous risks in traditional laminectomy operations, such as operations with a bone knife alone, which is likely to damage the nerve roots, spinal cord, and dura if not done properly, as well as the extent of intraoperative osteotomy relying entirely on operator experience. With the TINAVI robot, the extent of laminectomy is precisely planned in advance to obtain the best decompression with minimal trauma^21^. In the case of tuberculosis, accurate puncture of the lesion for multipoint sampling is possible with the assistance of the TINAVI robot to improve the positivity rate and to avoid damage to the adjacent vascular nerves^22^. Rational planning of lesion clearance at the later stage can reduce the risk of secondary surgery due to small lesion clearance and avoid damage to normal structures due to large clearance. Similarly, the TINAVI robot is also applicable to other infectious diseases of the spine.

Despite the great potential of the robot’s application, there are still many drawbacks: (1) the machine is expensive, but some domestic studies have concluded that it can shorten the total hospital stay and can yield significant social benefits^23^. (2) The size of the operating room is small, the robot is composed of more equipment with a larger size, the part in close contact with the patient requires strict aseptic protection, and the operator must plan the screw placement path several times during surgery. (3) The stability of the robot affects the accuracy of positioning. (4) Screw planning is greatly influenced by subjective operator factors, and different surgeons with different surgical experience make large errors in planning, which affects the accuracy. (5) When the joint surface of the articular eminence is rough and the soft tissue pressure is high, the planning of the screw path and deviation from the planned screw path may occur when the articular surface of the articulation is rough, the angle between the planned screw path and the vertebral body bone is narrow, and the tip of the guide pin can slip. We also lacked the ability to detect and correct slippage. To address these pain points, we believe that a suitable incision type should be selected and that the soft tissues should be adequately released when placing the access and screw. Second, the operator should select the guide pin, and the diameter of the guide pin should be closely matched to prevent excessive soft tissue tension or the embedded sleeve from affecting the tapping accuracy^24^. Finally, the actual screw path should be checked by lateral fluoroscopy during guide needle placement to see if it matches the planned screw path; our institution adopts the spine stapling method to monitor accuracy, which means that the screw entry point is placed at the vertex of the spine, and if it is not positioned correctly, the operator can distinguish it by visual inspection. If less than half of the guidewires are found to be poorly positioned intraoperatively, the robot can be reregistered and operated gently; and if more than half of the guidewires are poorly positioned, the robot can be checked for firm installation and obstruction of the optical tracking system and tracer. (6) The TINAVI robot has a long learning curve^25^, and for the initial contact with the robot, familiarity with system placement, image acquisition and screwing tract planning will increase the surgical time. Long segmental spinal lesions require multiple data acquisitions, and the preoperative planning of the surgical plan and “easy spine” freehand screwing combined with “difficult spine” robot-assisted screwing can reduce the operative time. (7) The position of the vertebral body may change after unilateral screwing. The lack of intraoperative real-time detection may lead to failure or poor screw placement on the opposite side, and excessive pressure of the guide on the underside should be avoided when the needle is drilled for placement, which may lead to a change in the vertebral body position. We usually use a Mayfield head frame to fix the head before cervical spine surgery. (8) The O-arm increases the radiation dose to the patient. (9) Previously built-in objects have had some effect on data acquisition, and the accuracy of revision surgery needs further study. (10) Intraoperative positioning and execution depend on the tracer, which often causes additional trauma, and we attach it to the table-side column to reduce trauma and reduce the impact of respiratory mobility.

There are some limitations to this study. As we are still in the early stages of use, the sample size was insufficient, and the number of patients needs to be increased at a later stage to enhance the credibility of the study findings. The retrospective study method will inevitably lose some clinical data, and other clinical data need to be analysed in further prospective studies with more comprehensive evaluation indices.

## Conclusion

The treatment of Levine–Edward type IIA (post-repositioning) hangman fractures with percutaneous pedicle screw fixation assisted by the TINAVI orthopaedic surgery robot can significantly improve screw placement accuracy with a low rate of invasion of the adjacent facet joint, a short operative time, a low bleeding rate, and high patient satisfaction. Although there are still many disadvantages, it still has good prospects for application.

## Data Availability

The datasets generated and/or analysed during the current study are available in the [fishare] repository, [10.6084/m9.figshare.24138357].
